# Measuring breast density: Comparing computer-automated breast density quantification with an observer-based method in a South African academic context

**DOI:** 10.4102/sajr.v22i2.1358

**Published:** 2018-08-21

**Authors:** Erica Prinsloo, Cornelia Minné, Wim Greeff

**Affiliations:** 1 Dr George Mukhari Academic Hospital, Pretoria, South Africa

## Abstract

**Background:**

Dense breast tissue may not only ‘mask’ small, non-calcified cancers but also represents an independent risk factor for the development of breast cancer. Computer-automated breast density quantification (CABD) software tools have been developed for the calculation of volumetric breast density.

**Objectives:**

This study sought: (1) to compare observer-based breast density scores, using the fifth edition of the Breast Imaging Reporting and Data System (BI-RADS), with the breast density scores calculated using CABD quantification software tools, (2) to determine inter-reader variability in breast density scoring between qualified radiologists, between radiologists in training (registrars) and between these two groups and (3) to determine intra-reader reliability in breast density scoring.

**Methods:**

A cross-sectional study was performed using the data of 100 patients (200 breasts). Three qualified radiologists and three registrars were asked to review the mammograms in question and to assign a breast density score according to the fifth edition of the Breast Imaging Reporting and Data System (BI-RADS) reporting system. Two readings took place at a minimum of 30 days apart. The percentage agreement between the automated and observer-based scores was calculated and intra-reader and inter-reader reliability values were determined.

**Results:**

The study found that there was poor agreement between the breast densities calculated by CABD and the more subjective observer-based BI-RADS density scores. These results further reflect a statistically significant degree of inter-reader and intra-reader variability in the evaluation of breast density.

**Conclusion:**

We conclude that the use of automated breast density quantification (i.e. CABD) is a valuable tool for the reduction of variability in breast density ratings.

## Introduction

Mammographic breast density has been the subject of scholarly scrutiny and legal debate for more than 40 years.^1^ Landmark studies by authors, such as Wolf and Tabár, paved the way for subsequent research regarding the relationship between mammographic density, parenchymal patterns and breast cancer risk.^1^

Published data suggest that dense breast tissue may not only ‘mask’ small, non-calcified cancers but also represents an independent risk factor for the development of breast cancer. A meta-analysis published in 2006^2^ showed that women with ≥ 75% dense breasts have a four to six times greater risk for developing breast cancer than do women with < 5% dense breasts. It is information like the above that has led to the introduction of wide-ranging legislation surrounding breast cancer screening in countries like the United States.^3^

Traditionally, breast density is assessed by using observer-based scores and qualitative area-based measurements. These methods, however, are limited in terms of subjectivity, reliability and reproducibility.^4^ More recently, computer-automated breast density quantification (CABD) software tools have been introduced for the calculation of volumetric breast density in an attempt to overcome these limitations.

Using these methods, women identified as having denser breasts would typically proceed to undergo additional screening with modalities such as automated breast ultrasound, handheld breast ultrasound or magnetic resonance imaging (MRI) to further evaluate for pathological lesions that may not be apparent on mammography.

### Breast cancer burden in South Africa and the role of mammography screening

Breast cancer is a leading cause of cancer-related deaths among females in the developing world.^5^ The South African incidence of breast cancer is 22.2% of all cancers with 29.99 adjusted cases per 10 000 per year.^6^

The five-year breast cancer survival rate in sub-Saharan Africa is estimated to be less than 40%, which compares unfavourably with the 86% rate of a country like the United States. Some authors ascribe the poor survival rates in sub-Saharan African patients to a lack of awareness, cultural beliefs and the existence of advanced disease stage at the time of presentation.^7^

As part of a standardised mammography report, the fifth edition of the American College of Radiology’s Breast Imaging Reporting and Data System (BI-RADS) initiative advises that breast density should be recorded and, where needed, described. Patients have to be informed regarding the diminished accuracy of mammography in dense breasts.^8^

The common aim of all mammography screening programmes is to reduce the rate of advanced breast cancer and to identify as many invasive lesions as early as possible. Mammography, as a screening tool, is limited in terms of detecting small pathological lesions in dense breasts. In spite of this limitation, however, mammography has been identified to date as the only modality capable of reducing breast cancer mortality.^9^ Thus, mammography remains the primary screening modality in patients with dense breasts, and supplemental screening efforts should be regarded as adjuncts to mammography.^10^ Supplemental screening options include automated breast ultrasound, handheld ultrasound, MRI and digital breast tomosynthesis.

### Assessment of mammographic density

Mammographic density refers to the ratio of radiopaque epithelial and stromal tissue elements in the background of radiolucent fatty tissue. The skin is also radiopaque and contributes to some of the mammographic density. Mammographic density does not correlate with breast firmness at physical examination.^4^

#### Observer-based assessment methods

The evaluation of breast density is usually performed by an experienced observer performing a visual assessment of a two-view mammogram (i.e. craniocaudal and mediolateral oblique views). Factors taken into account include the relative proportion of glandular tissue to fatty tissue in the breast, the shape and size of the breast, the fibroglandular pattern of the breast and the radiographic protocols that were used.^11^

The most widely used scoring system is the four-point, fifth edition of BI-RADS, with the following categories, depicted in Table 1.^8^

**TABLE 1:** Breast Imaging Reporting and Data System 5th edition breast density.

BI-RADS density category	Breast composition
A	The breasts are almost entirely fatty.
B	There are scattered areas of fibroglandular density.
C	The breasts are heterogeneously dense, which may obscure small masses.
D	The breasts are extremely dense, which lowers the sensitivity of mammography.

*Source*: D’Orsi CJ, Sickles EA, Mendelson EB. ACR BI-RADS Atlas. Breast Imag Report Data Syst. 2013:1–732

BI-RADS, breast imaging reporting and data system.

Density may also be scored on a continuous scale and expressed as a percentage.^11^

There remains; however, large inter-reader and intra-reader variability in the observer-based evaluation of breast density. Some authors have suggested an inter-reader agreement of only 49%.^4^ Observer-based scoring is still the most widely used method because of the additional costs involved with computer-based assessments.^12^

#### Computer-based qualitative methods

Vendor-specific digital software algorithms can calculate reproducible breast densities and express them in terms of either area density or volume density.

#### Area density percentage algorithms

Interactive thresholding is a semi-automated method of area density calculation.^12^ This method relies on the user to select the grey level threshold value for a digital mammogram and retains a measure of subjectivity.^12^

#### Volume density percentage algorithms

Volume density percentages can be calculated from the three-dimensional data sets acquired by digital breast tomosynthesis, ultrasound, computed tomography (CT) or MRI.

Volume density percentage is defined as:

Vf=Vf/Vt×100 [Eqn 1]

where *Vf* is the fibroglandular tissue volume and *Vt* is the total breast volume.^12^

Alternatively, two-dimensional digital mammograms can be used to calculate the three-dimensional properties of the breast. This is computed by factoring in the image pixel data, as well as radiographic protocol elements such as X-ray tube potential, target material, filter material, paddle height and breast compression.^11^ These algorithms may help to reduce observer errors; however, considerable miscalculations are still possible because of the fact that the three-dimensional properties of the breast are evaluated using two-dimensional images.

Currently, there are two United States food and drug administration-approved software programs available that provide fully automated volume density percentages, each with their own proprietary algorithms: Quantra (Hologic Inc., Bedford, MA, USA) and Volpara (Mātikana International, Wellington, New Zealand).^12^

## Research methods

### Study design

This study employed a cross-sectional design and evaluated CABD against an observer-based BI-RADS fifth edition breast density scoring system. Patients who underwent full-field digital mammography between 01 October 2015 and 31 July 2016 at the Dr George Mukhari Academic Hospital (DGMAH) in Ga-Rankuwa, South Africa, for both screening and diagnostic purposes, were included in this study. The majority of patients presenting to the mammography unit were symptomatic patients referred on the grounds of their clinical findings.

The patients, selected for participation in this study included all asymptomatic female patients who presented for screening mammograms at the DGMAH; those symptomatic patients aged 18 years or older who were referred to the DGMAH with the clinical suspicion of breast cancer; and patients who had undergone the standard craniocaudal and mediolateral oblique views. Conversely, those excluded from this study were patients who had undergone a previous mastectomy, patients with ulcerating breast cancer and patients with incomplete data.

### Data collection

Three qualified radiologists and three registrars (radiology residents) were asked to review the mammograms in question and assign a breast density score to each patient according to the BI-RADS fifth edition reporting system.

The specialists were all general radiologists with an interest in mammography whose experience ranged from four years to newly qualified. The registrars’ experience ranged from two to six months of full-time rotation in mammography.

The readers were specifically asked not to interpret pathology and were blinded to the automated breast density score. The readers were asked not to change the window level of the images.

To determine intra-reader variability, each mammogram was scored twice by each reader. Readings took place at a minimum of 30 days apart. Readers were blinded to their previous scores, as well as to the scores of other readers.

The computer-generated breast density results were recorded by a research assistant. The typical information values calculated by the CABD are presented in Table 2.

**TABLE 2:** The typical information values calculated by the computer-aided diagnosis.

Symbol	Definition
Vfg (cm^3^)	Volume of fibroglandular tissue, in cubic cm.
Vb (cm^3^)	Volume of breast, in cubic cm.
Vbd (%)	Volume of breast density, in percentage of total breast volume.
Abd (%)	Area of breast density, in percentage of total breast area.
Vbd-score	How an individual woman’s Vbd compares with a reference population.
Vfg-score	How an individual woman’s Vfg compares with a reference population.
Q-abd	A BI-RADS-like integer score of breast composition.
q-abd	A BI-RADS-like fractional score of breast composition.

BI-RADS, breast imaging reporting and data system; cm, centimetre; cm^3^, cubic centimetre

A Dimensions 8000 with SVDX 400 workstation and Quantra Version 2.1.1 software package (both Hologic Inc., Bedford, MA, USA) were used.

### Statistical analysis

Comparisons of the automated breast density and volume with the observer-based scoring were assessed by calculation of a percentage agreement.

Inter- and intra-reader reliabilities were assessed by calculation of kappa statistics and correlations. Categorical scores were compared using Fisher’s exact test. All statistical procedures were performed via Statistical Analysis System (SAS Institute Inc., Cary, NC, USA), Release 9.4, running on Microsoft Windows^®^ (Microsoft Corp., Redmond, WA, USA) for a personal computer. All statistical tests were two-sided, and *p*-values smaller than or equal to 0.05 were considered to be significant.

Various arbitrary guidelines exist to characterise kappa values. Fleiss characterises kappa values of more than 0.75 to be excellent, those from 0.4 to 0.75 as fair to good and those below 0.4 as poor.

A sample size of 100 patients (200 breasts) was used.

## Results

The mean age of the screened population group was 51.7 years (with a ± standard deviation of 13.89 years). The median age was 51 years (interquartile range: 41–63 years). The youngest patient was 18 years of age and the oldest patient was 84 years of age.

It is important to note that symptomatic patients under the age of 35 years only underwent mammography after ultrasound was performed, when calcifications were suspected. Limited views are usually done, as per institutional protocols. Standard craniocaudal and mediolateral views in patients under the age of 35 years are only employed for specific indications and where deemed necessary by the reporting radiologist.

On comparing the BI-RADS density category scores (a–d) by the readers with those of the CABD, the study found that there was a 36.7% agreement (95% confidence interval [CI]: 34.0%–39.5%) for the registrar group and only a 33.2% (95% CI: 30.5% – 35.9%) for the specialist group. When using a dichotomous score of dense (BI-RADS scores c and d) versus non-dense (BI-RADS scores a and b), these scores improved slightly to 57.8% (95% CI: 55.0% – 60.6%) for the registrar group and 51.2% (95% CI: 48.3% – 54.0%) for the specialist group, respectively.

The intra-reader reliability (comparing first and second readings) using the BI-RADS density category scores (a–d) for the registrar group was 57% (kappa value = 0.4012 and *p* < 0.0001). The intra-reader reliability for the specialist group was 74.1% (kappa value = 0.6012 and *p* < 0.001). When using the dichotomous score of dense (BI-RADS scores c and d) versus non-dense (BI-RADS scores a and b), the intra-reader reliability improved to 70.9% (kappa value = 0.3940%) for the registrars and 88.0% (kappa value = 0.6799) for the specialists.

The inter-reader reliability for both groups was found to be mostly poor. These findings are presented in Tables 3 and 4.^13^ The readers were anonymised and assigned a reader code.

**TABLE 3:** Inter-reader reliability for density by registrars.

Reader code	First reading (simple kappa)	Second reading (simple kappa)
A by C	0.31	0.08
A by B	0.45	0.38
C by B	0.23	0.23

Note: Various arbitrary guidelines exist to characterise kappa values. Fleiss characterises kappa values over 0.75 as excellent, 0.4–0.75 as fair to good and below 0.4 as poor.^13^

**TABLE 4:** Inter-reader reliability for density by specialists.

Reader code	First reading (simple kappa)	Second reading (simple kappa)
D by E	0.46	0.28
D by F	0.48	0.27
E by F	0.61	0.60

Note: Various arbitrary guidelines exist to characterise kappa values. Fleiss characterises kappa values over 0.75 as excellent, 0.4–0.75 as fair to good and below 0.4 as poor.^13^

## Ethical considerations

Informed consent was not needed for this type of retrospective study because data were anonymised and the study did not present an extra burden to the patients and their families.

Written consent was obtained from hospital management and a clearance certificate was secured from the relevant medical school and university ethics committees prior to the commencement of the study (SMUREC/M/240/2016: PG).

## Discussion

Our study found that there was poor agreement between the breast densities calculated by CABD and the more subjective observer-based BI-RADS density scores. In comparison, a recent large European study^14^ demonstrated moderate agreement between radiologists using the BI-RADS fourth edition and CABD measurements using Volpara software. The agreement between categorical volumetric density and BI-RADS scores in the above-mentioned study was 57.1% (kappa 0.55 [0.53–0.56]).

Our findings of poor agreement between the CABD and observers in terms of breast density could be attributed to various factors such as a bias towards defensive practice, a tendency to over or underestimate, work pressure and fatigue, viewing conditions and contextual influences.^12^ It is also important to remember that the specialist group consisted of radiologists with less than five years of experience. Furthermore, these radiologists were all generalists and not dedicated breast imagers. It could be argued that this is representative of the actual situation in many South African academic institutions.

On the contrary, the available software for fully automated breast density measurements has been shown to be robust and be able to provide reproducible quantitative measures.^15,16^

Our results further reflect the presence of a statistically significant degree of inter- and intra-reader variability in the evaluation of breast density. These findings are consistent with those of other large studies.^17,18^

In a recent multicentre observational study^19^ that included data from 200 000 screening mammograms, the rate of individual radiologists assigned to a dichotomous ‘dense category’ ranged widely from 6.3% to 84.5%. When consecutive mammograms were interpreted by different radiologists, over an average span of 1.2 years, there was 17.2% discordance in the ‘dense’ versus ‘non-dense’ assignments.

It is furthermore important to note that breast density was characterised in the BI-RADS fourth edition on the basis of the percentage of glandular tissue within each breast (i.e. into the categories of < 25%, 25% – 50%, 51% – 75% or > 75%, respectively). In the new fifth edition, these percentiles are eliminated and replaced by the four category descriptors.

The advent of the fifth edition of BI-RADS also appears to have had an additional deleterious impact on the inter-reader agreement of breast density scoring, in comparison with the BI-RADS fourth edition.^20^

It is argued that, because of the subjective nature of the BI-RADS breast density assessment, the decision for supplemental screening may be more dependent on who the reporting radiologist is than on the actual amount of fibroglandular tissue in the imaged breast.^21^

The call is being made for automated breast density measures to be adopted as part of the standard mammographic evaluation protocol in order to reduce variability in breast density ratings and to produce standardised thresholds for supplementary screening.

Currently, there is no formal mammography screening programme in South Africa. Mammography screening currently takes place on an individual case-by-case basis.

### Limitations of the study

Notably, the available specialists who took part in this study all had less than five years of experience. This is; however, a realistic reflection of the situation in many South African academic hospitals. Of the mammograms that were used, 66% were normal, 26% had masses and 8% had other findings such as architectural distortion, calcifications and oedema. It is unclear if visible lesions on these images may have produced a bias towards denser scoring by the readers. A future study using normal mammograms may be of benefit.

## Conclusion

The authors are of the opinion that the use of automated breast density quantification is a valuable tool to reduce variability among breast density ratings. This may be especially true in the South African academic context wherein preliminary mammography reports are generated by trainees and approved by general radiologists, who often have less than five years of experience.

The potential benefits and costs of CABD use in South African academic hospitals should be carefully considered.

There is a need for the development of national protocols regarding the use of software tools in the evaluation of breast density to occur. Such protocols should also direct decision-making efforts towards adding supplementary screening for women with dense breasts. This; however, should ultimately form part of a larger drive towards the implementation of a South African national breast screening programme.

## References

[1] Wolf JN Risk for breast cancer development determined by mammographic parenchymal pattern. Cancer. 1976;37:2486–2492. 10.1002/1097-0142(197605)37:5<2486::AID-CNCR2820370542>3.0.CO;2-8 1260729

[2] Pettersson A, Graff RE, Ursin G, et al Mammographic density phenotypes and risk of breast cancer: A meta-analysis. J Natl Cancer Inst. 2014;106(5):dju078 10.1093/jnci/dju078 24816206 PMC4568991

[3] Jennifer S, Haas CPC The divide between breast density notification laws and evidence-based guidelines for breast cancer screening. JAMA Intern Med. 2015;175(9):1439–1440. 10.1001/jamainternmed.2015.3040 26147642 PMC4564307

[4] Freer PE Mammographic breast density: Impact on breast cancer risk and implications for screening. Radiographics. 2015;35:302–315. 10.1148/rg.352140106 25763718

[5] Torre LA, Bray F, Siegel RL, Ferlay J, Lortet-Tieulent J, Jemal A Global cancer statistics, 2012. CA Cancer J Clin. 2015;65(2):87–108. 10.3322/caac.21262 25651787

[6] National Cancer Registry (NCR) of South Africa 2013 http://www.nicd.ac.za/wp-content/uploads/2017/03/2013NCR.pdf

[7] Cumber NS, Nchanji KN, Tsoka-Gegweni JM Breast cancer among women in sub-Saharan Africa: Prevalence and a situational analysis. Southern Afr J Gyneacol Oncol. 2017;9(2):35–37. 10.1080/20742835.2017.1391467

[8] D’Orsi CJ, Sickles EA, Mendelson EB ACR BI-RADS Atlas. Breast Imag Report Data Syst. 2013:1–732.

[9] Tabár L, Vitak B, Chen TH-H, et al Swedish two-county trial: Impact of mammographic screening on breast cancer mortality during 3 decades. Radiology. 2011;260(3):658–663. 10.1148/radiol.11110469 21712474

[10] Ray KM, Price ER, Joe BN Breast density legislation: Mandatory disclosure to patients, alternative screening, billing, reimbursement. Am J Roentgenol [serial online]. 2015 [cited 2017 Nov 2];204(2):257–560. Available from: http://www.ajronline.org/doi/10.2214/AJR.14.13558 10.2214/AJR.14.13558 25615746

[11] Morrish OWE, Tucker L, Black R, Willsher P, Duffy SW, Gilbert FJ Mammographic breast density: Comparison of methods for quantitative evaluation. Radiology. 2015;275(2):356–365. 10.1148/radiol.14141508 25559234

[12] Winkler NS, Raza S, Mackesy M, Birdwell RL Breast density: Clinical implications and assessment methods. RadioGraphics. 2015;35:316–324. 10.1148/rg.352140134 25763719

[13] Fleiss JL, Myunghee BL, Paik C Statistical methods for rates and proportions 3rd ed. Hoboken, New Jersey: John Wiley & Sons, Inc.; 2003.

[14] Sartor H, Lang K, Rosso A, Borgquist S, Zackrisson S, Timberg P Measuring mammographic density: Comparing a fully automated volumetric assessment versus European radiologists: A qualitative classification. Eur Radiol. 2016;26(12):4354–4360. 10.1007/s00330-016-4309-3 27011371 PMC5101269

[15] Brandt KR, Scott CG, Ma L, et al Comparison of clinical and automated breast density measurements: Implications for risk prediction and supplemental screening. Radiology. 2016;279(3):710–719. 10.1148/radiol.2015151261 26694052 PMC4886704

[16] Keller BM, Nathan DL, Wang Y, et al Estimation of breast percent density in raw and processed full field digital mammography images via adaptive fuzzy c-means clustering and support vector machine segmentation. Med Phys [serial online]. 2012 Aug [cited 2018 Mar 23];39(8):4903–4917. Available from: http://www.ncbi.nlm.nih.gov/pubmed/22894417 10.1118/1.4736530 PMC3416877 22894417

[17] Ooms EA, Zonderland HM, Eijkemans MJ, et al Mammography: Interobserver variability in breast density assessment. Breast. 2007;16(6):568–576. 10.1016/j.breast.2007.04.007 18035541

[18] Ciatto S, Bernardi D, Calabrese M, et al A first evaluation of breast radiological density assessment by QUANTRA software as compared to visual classification. Breast. 2012;21(4):503–506. 10.1016/j.breast.2012.01.005 22285387

[19] Sprague BL, Conant EF, Onega T, et al Variation in mammographic breast density assessments among radiologists in clinical practice. Ann Intern Med [serial online]. 2016 Oct 4 [cited 2017 Nov 2];165(7):457 Available from: http://annals.org/article.aspx?doi=10.7326/M15-2934 10.7326/M15-2934 PMC5050130 27428568

[20] Irshad A, Leddy R, Ackerman S, et al Effects of changes in BI-RADS density assessment guidelines (Fourth Versus Fifth Edition) on breast density assessment: Intra- and interreader agreements and density distribution. Am J Roentgenol [serial online]. 2016 Dec [cited 2018 Apr 13];207(6):1366–1371. Available from: http://www.ajronline.org/doi/10.2214/AJR.16.16561 10.2214/AJR.16.16561 27656766

[21] Gastounioti A, Conant EF, Kontos D Beyond breast density: A review on the advancing role of parenchymal texture analysis in breast cancer risk assessment. Breast Cancer Res. 2016;18(1):1–13. 10.1186/s13058-016-0755-8 27645219 PMC5029019

